# A data management and publication workflow for a large-scale, heterogeneous sensor network

**DOI:** 10.1007/s10661-015-4594-3

**Published:** 2015-05-13

**Authors:** Amber Spackman Jones, Jeffery S. Horsburgh, Stephanie L. Reeder, Maurier Ramírez, Juan Caraballo

**Affiliations:** Utah Water Research Laboratory, Utah State University, 8200 Old Main Hill, Logan, UT 84322-8200 USA; Department of Civil and Environmental Engineering and Utah Water Research Laboratory, Utah State University, 8200 Old Main Hill, Logan, UT 84322-8200 USA

**Keywords:** Cyberinfrastructure, Sensor, Quality control, Data management, Hydrology, Data models, Observatory

## Abstract

It is common for hydrology researchers to collect data using in situ sensors at high frequencies, for extended durations, and with spatial distributions that produce data volumes requiring infrastructure for data storage, management, and sharing. The availability and utility of these data in addressing scientific questions related to water availability, water quality, and natural disasters relies on effective cyberinfrastructure that facilitates transformation of raw sensor data into usable data products. It also depends on the ability of researchers to share and access the data in useable formats. In this paper, we describe a data management and publication workflow and software tools for research groups and sites conducting long-term monitoring using in situ sensors. Functionality includes the ability to track monitoring equipment inventory and events related to field maintenance. Linking this information to the observational data is imperative in ensuring the quality of sensor-based data products. We present these tools in the context of a case study for the innovative Urban Transitions and Aridregion Hydrosustainability (iUTAH) sensor network. The iUTAH monitoring network includes sensors at aquatic and terrestrial sites for continuous monitoring of common meteorological variables, snow accumulation and melt, soil moisture, surface water flow, and surface water quality. We present the overall workflow we have developed for effectively transferring data from field monitoring sites to ultimate end-users and describe the software tools we have deployed for storing, managing, and sharing the sensor data. These tools are all open source and available for others to use.

## Introduction

Advances in the development of in situ environmental sensors have led to the ubiquitous use of sensors and sensor networks in environmental monitoring (Martinez et al. 2004; Hart and Martinez 2006; Rundel et al. 2009). Researchers and practitioners are collecting data with in situ sensors at high frequencies, for extended durations, and with spatial distributions that generate volumes of data for which the deployment of cyberinfrastructure (CI) for data management is necessary. Additional challenges are presented by networks that consist of multiple data collection sites, sensors, and personnel (Rüegg et al. 2014). Consistency in data management across these factors can facilitate data integration.

CI integrates computing hardware, digitally enabled sensors, data observatories and experimental facilities, interoperable software and middleware service and tools, and data and networks (National Science Foundation 2007). Researchers and practitioners that are operating environmental observatories and sensor networks need CI tools for data import and storage as well as data discovery, access, and distribution (Muste et al. 2010, 2013; Mason et al. 2014). In addition to addressing challenges presented by the sheer quantity of data, monitoring networks need practices to ensure high data quality, including procedures and tools for post-processing (e.g., Steiner et al. 1999; Foken et al. 2005; Collins et al. 2006; Campbell et al. 2013). Data quality is further enhanced if data managers and users are able to relate the physical infrastructure used for measurements and site and equipment maintenance events to the observational data generated by the sensors.

In this paper, we present a workflow for management and publication of in situ sensor data observed within an environmental sensor network. In particular, we describe the practical implementation of the workflow using readily available software and CI tools. Here, we work toward integrating software tools to support data management with mechanisms to facilitate data publication and discovery, an area that Mason et al. (2014) identify as a gap in research. We have also worked toward automation of the workflow, as Ruddell et al. (2014) point out that any manual component of a data flow is costly in time and resources.

We present the workflow in the context of a case study of the innovative Urban Transitions and Aridregion Hydrosustainability (iUTAH) Gradients Along Mountain to Urban Transitions (GAMUT) environmental observatory. The iUTAH GAMUT monitoring network consists of aquatic and climate sensors deployed in three watersheds to monitor gradients as water transitions from high elevation snowmelt through downstream urban and urbanizing areas. The variety of environmental sensors and the multi-watershed, multi-institutional nature of the GAMUT network necessitate a well-planned and efficient workflow for acquiring, managing, and sharing sensor data. The workflow was developed and successfully implemented for this case. Other networks may have slightly different needs for which the workflow could be easily adapted. In “Background and related work,” we describe background and related work. The section “Case study: the iUTAH GAMUT network” describes our use case, the iUTAH GAMUT sensor network. In “Workflow requirements,” we describe the requirements that we have defined for CI to meet the needs of this and similar monitoring networks. The section “Data management and publication workflow” describes the overall workflow applied to meet the requirements, with details on the specific components that have been developed and implemented in the workflow.

## Background and related work

Required functionality of CI for sensor data management includes the ability to store and version data series, perform quality control processing, track equipment deployments, calibrations, and other events related to monitoring site maintenance, and to link this information to the observational data being collected (Chave et al. 2009; Horsburgh et al. 2011; Porter et al. 2012; ESIP EnviroSensing Cluster 2014). All of this is imperative to ensure the quality and utility of sensor-based data products. Commercial software systems are available with good functionality, but their cost can be out of reach for many small research groups, some are tied to specific instrument/equipment manufacturers limiting their general applicability, and none currently interface with a long-term community data archive to assist scientists in meeting the data management, sharing, and archival requirements of funding agencies such as the National Science Foundation (e.g., http://www.nsf.gov/bfa/dias/policy/dmp.jsp). The ESIP EnviroSensing Cluster (2014) provides a description of commercially available and open source software programs that address aspects of sensor data management.

A number of open source tools have been developed for research groups and sites conducting long-term monitoring using in situ sensors. The Consortium of Universities for the Advancement of Hydrologic Science, Inc. (CUAHSI) Hydrologic Information System (HIS) HydroServer software stack (Horsburgh et al. 2009, 2010) is a viable, open source solution and is currently being used by many research groups across the country. The software components of the HydroServer software stack use web services and standards to interface with the CUAHSI Water Data Center (https://wdc.cuahsi.org), which is a new facility funded by the National Science Foundation that supports data access and publication for the hydrologic sciences and other scientific communities.

Indeed, several authors have described a general architecture and components of CI for managing environmental sensor data in the context of environmental observatories using the CUAHSI HIS. Horsburgh et al. (2011) describe components of an integrated observatory information system, including infrastructure for (1) observation and communication; (2) data storage and management; (3) data quality assurance, control, and provenance; (4) data publication and interoperability; and (5) data discovery and presentation. They provide a case study that uses components of the CUAHSI HIS to support these functionalities. Conner et al. (2013) describe a simplified version of the CUAHSI HIS HydroServer software stack that can be implemented by research labs and groups for archiving and sharing environmental observations data. Muste et al. (2010, 2013) describe a CI for supporting integrated water resource management with similar use of the CUAHSI HIS components to support management of environmental sensor data. Muste et al. (2010) point out that a system built around the CUAHSI HIS has the benefit of broad community support and facilitates implementation of data management to support science in local observatories. Mason et al. (2014) and Izurieta et al. (2011) describe a system for sensor data management within ecological research laboratories. Their system implements a custom data model for data storage and custom built software components for data editing and quality control. However, they use CUAHSI HIS components, in particular the Observations Data Model (ODM) (Horsburgh et al. 2008) and the WaterOneFlow/WaterML web services (Zaslavsky et al. 2007), as an interoperability mechanism for ultimately publishing the data via the CUAHSI HIS.

In this paper, we present the sensor data management and publication workflow and focus specifically on its physical implementation and automation using the CUAHSI HIS, aspects that have not been well described by other authors. We describe in detail how components of the CUAHSI HIS were implemented within an actual case study for managing and publishing environmental sensor data—the iUTAH GAMUT network. As one of the major goals of the CI supporting the GAMUT network was to publish the data in such a way that it can be archived at the CUAHSI Water Data Center, this paper focuses on the details of how this can be done, demonstrating how others can adopt similar methods for managing their sensor data and interfacing with the CUAHSI Water Data Center.

## Case study: the iUTAH GAMUT network

**Fig. 1:**
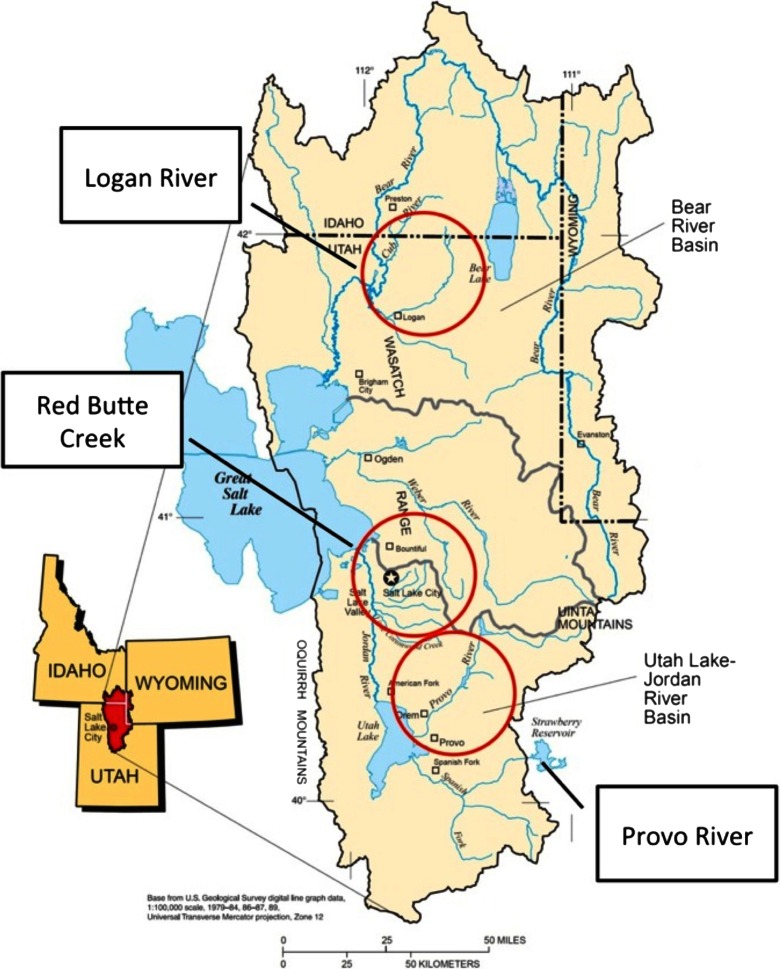
Location of the three watersheds in the iUTAH GAMUT network. Adapted from Baskin et al. (2002)

iUTAH (http://iutahepscor.org) is an interdisciplinary research project studying water sustainability in Utah, USA, and is particularly focused on the impact of transitioning urban areas on water availability, use, and quality. A major component of iUTAH is the deployment of the GAMUT ecohydrologic observatory in three watersheds in northern Utah: the Logan River, Red Butte Creek, and the Provo River (Fig. 1). All three watersheds share common water sources in winter snowpack that accumulates in forested mountain headwaters. That water is used in the spring and summer in downstream urban or urbanizing valleys, which differ between the three watersheds in the level of urbanization that has taken place. The Logan River watershed is rapidly transitioning from agricultural to urban land use; Red Butte Creek contains an established urban area in Salt Lake City; and the Provo River is gradually transitioning from agricultural to exurban development.

**Fig. 2:**
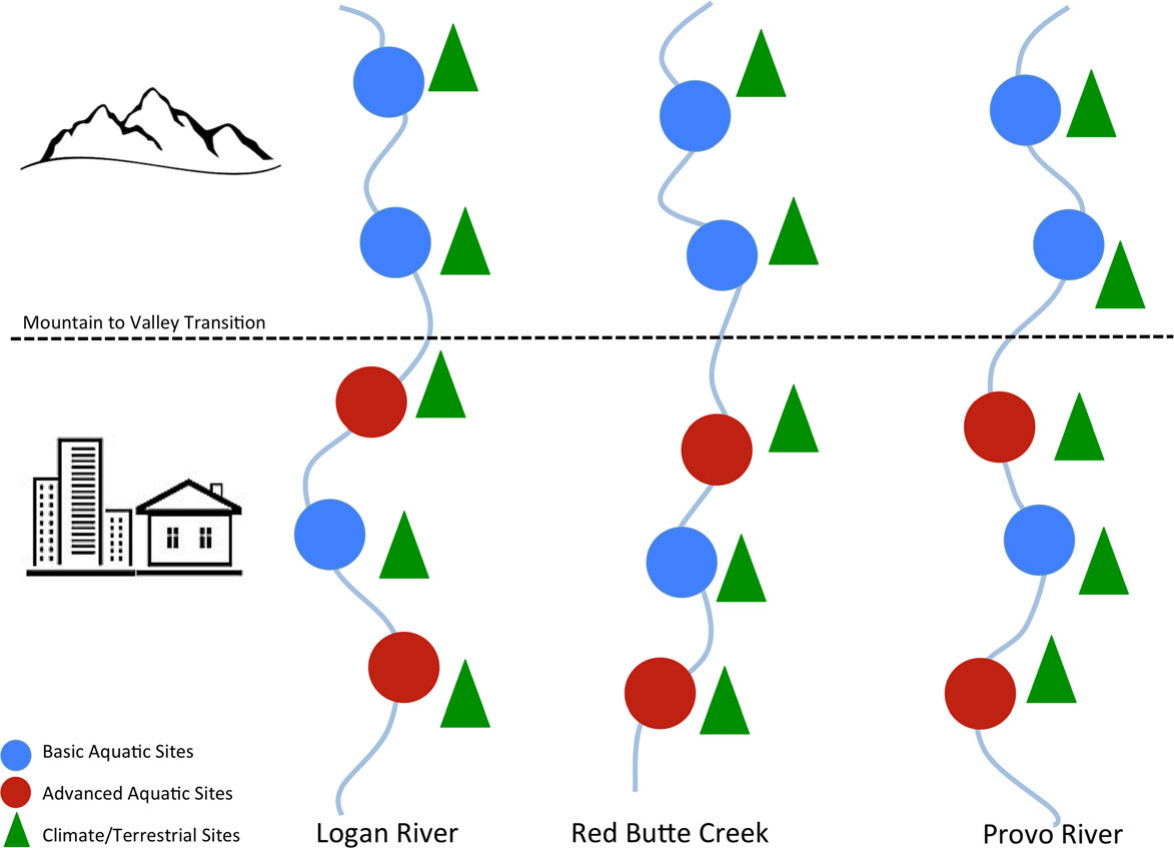
Conceptual configuration and siting of monitoring locations the three GAMUT watersheds

**Fig. 3:**
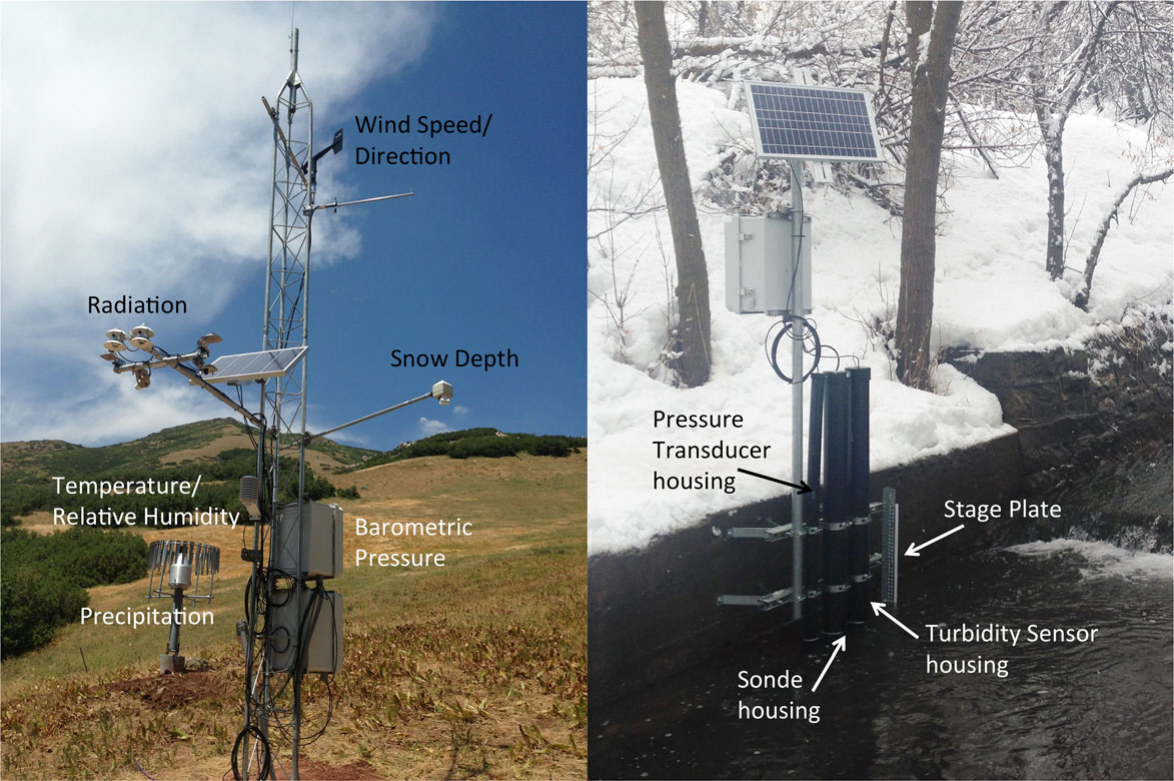
Configuration of typical GAMUT climate and aquatic sites

The long-term objectives of the network include better understanding and prediction of the effects of climate and land use change on the water budget and water quality within both the high elevation forest ecosystems and the urbanizing areas (http://gamut.iutahepscor.org). Toward these ends, GAMUT consists of a network of aquatic and terrestrial sites having in situ sensors collecting continuous, high frequency data on weather, energy balance, precipitation, snow accumulation, soil moisture, surface water flow, and surface water quality. Multiple sites of each kind have been deployed within each watershed to capture both mountain and valley areas (Fig. 2). Table 1 contains a list of sensors and the variables being measured at typical GAMUT sites, and typical GAMUT sites are shown in Fig. 3. At the time of this writing, the GAMUT Network consisted of 14 aquatic sites and 14 terrestrial sites. A total of 1300 time series for 121 variables are being recorded, and over 43,400,000 individual data values have been recorded or produced through the data quality control versioning process. The GAMUT network is a collaborative effort between researchers at Utah State University (USU), the University of Utah (UofU), and Brigham Young University (BYU). Each university built the monitoring infrastructure in their nearby watershed, and each university employs a full-time watershed technician to manage the infrastructure.

**Table 1:** Sensors and associated variables being collected at typical aquatic and climate sites in the GAMUT network

Site type	Sensor manufacturer and name	Output variables
All sites	Campbell Scientific, Inc. Datalogger (CR800, CR1000, CR3000)	Datalogger panel temperature, scan counter, battery voltage
	Campbell Scientific, Inc. CS210	Enclosure relative humidity
	Campbell Scientific Inc. 18166	Enclosure open door counter
Basic aquatic sites	YSI, Inc. EXO 599870-01	Water temperature, specific conductance
	YSI, Inc. EXO 599702	pH
	YSI, Inc. EXO 599100-01	Dissolved oxygen (mg/L), dissolved oxygen (% of saturation), dissolved oxygen (local % of saturation)
	YSI, Inc. EXO Sonde	Sonde output time stamp, power delivered to sonde
	Forest Technology Systems, Inc. DTS-12	Average turbidity, median turbidity, minimum turbidity, maximum turbidity, turbidity variance, best easy systematic turbidity, water temperature, sensor wiper indicator
	Campbell Scientific, Inc. CS450	Gage height, water temperature, sensor NaN counter, gage height offset
Advanced aquatic sites	YSI, Inc. EXO 599102	Blue-green algae
	YSI, Inc. EXO 599102	Chlorophyll fluorescence
	YSI, Inc. EXO 599104	Fluorescent dissolved organic matter
Standard climate sites	Campbell Scientific, Inc. HC2S3	Average air temperature, minimum air temperature, maximum air temperature, relative humidity
	Campbell Scientific, Inc. CS210	Dew point temperature, vapor pressure
	Campbell Scientific, Inc. CS106	Barometric pressure
	R.M. Young 05303-45	Average wind speed, average wind direction, maximum wind direction, wind direction standard deviation
	Geonor Inc. TB-200	Total precipitation, frequency of wire vibration, precipitation sensor offset, precipitation hourly difference, heater indicator counter, sensor inlet temperature
	Judd Communications LLC DS	Average snow depth, snow depth offset, snow depth measurement counter, air temperature
	Hukseflux NR01	Incoming shortwave radiation, outgoing shortwave radiation, incoming longwave radiation (corrected), outgoing longwave radiation (corrected), incoming longwave radiation (uncorrected), outgoing longwave radiation (uncorrected), net radiation, sensor temperature
	Apogee Instruments, Inc. SP-230	Incoming shortwave radiation
	Apogee Instruments, Inc. SQ-110	Incoming photosynthetically active radiation, outgoing photosynthetically active radiation
	Hukseflux NR01/Apogee Instruments, Inc. SP-230	Heater on/off indicator
	Campbell Scientific, Inc. CS210/NR01	Temperature difference to activate heater
	Apogee Instruments, Inc. SI-111	Terrestrial surface temperature, radiometer sensor temperature, slope for temperature calculation, intercept for sensor calculation, radiometer voltage output
	Acclima, Inc. ACC-SEN-SDI	Volumetric water content, soil temperature, bulk electrical conductivity, permittivity. All at 5-, 10-, 20-, 50-, and 100-cm soil depths
	Apogee Instruments, Inc. ST110	Average air temperature, maximum air temperature, minimum air temperature, aspirated radiation shield rotation
	Combination of sensors	Tall grass reference evapotranspiration, short grass reference evapotranspiration

## Workflow requirements 

The multi-site, multi-watershed, and multi-institution nature of the GAMUT network presented challenges to the implementation of CI including the following: providing access to data for different levels of users at various stages of data collection and processing, tracking the deployment and maintenance of physical monitoring equipment, and managing the data consistently across three watersheds and major organizations. These requirements are not unlike those faced by many other large data collection efforts in the context of environmental observatories. The following sections describe the requirements that drove the design of our data management workflow for the GAMUT network.

### Automated workflow process

In situ sensors are often advertised as autonomous and low maintenance. However, when deployed as part of a monitoring network, they require cleaning, calibration, maintenance of telemetry connections and power supply, regular data verification, troubleshooting, and data post-processing for quality control. These are all time consuming for network personnel. To efficiently use the available time of researchers, field technicians, and data managers, an objective was to automate as many steps in the workflow as possible. Automating the transfer of data and facilitating the entry of metadata also serve to reduce potential errors in data and metadata (Vivoni and Camilli 2003; White et al. 2009; Campbell et al. 2013).

### Data available in near real-time via the Internet

Data from in situ sensors need to be accessed by various users for different purposes such as research, climate and water quality modeling, education (k-12 and higher education), and network maintenance. The variety of users, both in experience (e.g., researchers, students, local agency partners, technicians, etc.) and in geography, necessitates an Internet-based platform that does not require specialized software for broad access to and visualization of the data. This is important functionality identified by Horsburgh et al. (2011), Muste et al. (2013), and Demir and Krajewski (2013). Some applications, such as planning for field sampling efforts and quality assurance checks of the data, require access to current conditions. In the case of GAMUT, we also wanted the data to be available on the Internet using the standard metadata descriptions, web service interfaces, and data formats required for the data to be published via the CUAHSI HIS.

### Automated quality assurance

Many analyses and models demand continuous records. As recommended by Campbell et al. (2013), efforts were made in the design of the GAMUT monitoring sites to ensure that each site used robust components, had adequate power supply, and included onsite data storage. In some cases, GAMUT sites even include redundant sensors. These precautions were designed to prevent hardware and telemetry system-related data loss. We also developed a quality assurance/quality control (QA/QC) plan at the outset of data collection to document the standard practices we adopted. Active quality assurance is required to ensure that data loss from malfunctions and failures are minimized and that the data collected are of high quality. This includes site visits and maintenance, as well as regular and thorough visual inspection of the data. However, the number of sites and data streams in many sensor networks like GAMUT is too great to permit thorough, daily, visual inspection. Thus, an effective workflow needs to implement automated data checking and notifications of potential problems to technicians.

### Standardized and traceable quality control

Initial design of the GAMUT network involved a consistent suite of sensors at each site (aquatic and terrestrial), with a standard sensor installation and consistent programming of sensors and dataloggers between sites so that the resulting data are as comparable as possible. However, after data are collected, post-processing steps for quality control must be taken, a task that is managed by different people in each watershed. Despite our best efforts to standardize data collection infrastructure, the ultimate comparability of data between watersheds also depends on consistent quality control across technicians, watersheds, and sites. Traceability and reproducibility of edits made in the quality control process are also important requirements for preserving the provenance of the data. This need has also been identified by Campbell et al. (2013) and Rüegg et al. (2014).

### Data versioning and archival

Despite efforts to ensure high data quality, field data are noisy and contain gaps and potential errors and anomalies. In all cases, the raw data need to be maintained and made accessible (Campbell et al. 2013). However, copies of the data may be created as quality control is performed. Additional versions of data may be created as users aggregate, process, or derive quality-controlled data into new, higher-level products. Thus, data versioning had to be built into the workflow. This need has been recognized by others in the management of sensor data (e.g., White et al. 2013; Mason et al. 2014). We adopted as requirements the following strategies/best practices suggested by the ESIP EnviroSensing Cluster (2014) to increase the longevity and interoperability of the GAMUT data: (1) publishing periodic snapshots of the continuous time series; (2) assignment of persistent identifiers to published files; (3) maintaining explicit data versions; and (4) adoption of appropriate platform and software independent data storage formats for archival.

### Data linked to field information

A step often overlooked in the management of sensor data is tracking of the inventory of physical infrastructure (e.g., sensors, dataloggers, solar panels, etc.) and related field activities such as site visits, calibrations, and other maintenance. For large-scale sensor networks, these details may be recorded cursorily when they occur in field notebooks or field sheets, but may be managed in a dispersed manner (e.g., multiple notebooks or field sheets managed by multiple technicians). These details often become an important reference when subsequently processing, interpreting, and analyzing the data. The physical equipment used for data collection can have drastic effects on resulting data, particularly when failures occur. It is not uncommon for environmental time series resulting from sensor measurements to span multiple sensor maintenance and calibration periods and even multiple sensor deployments. Information describing maintenance, calibrations, swapping components, etc. recorded in field notebooks or static files is rarely linked directly to the data observed by these instruments in a way that it could be used to evaluate and interpret results. Yet, there are many scenarios when performing post-processing and quality control or even eventual analyses of the data that require consultation of the record of field activities (Izurieta et al. 2011; ESIP EnviroSensing Cluster 2014). The desire to more closely relate observations and research results with descriptive metadata and provenance information is behind a recent push toward electronic field and laboratory notebooks (e.g., Weng et al. 2012; Zaki et al. 2013; Wolniewicz 2014) as well as efforts by sensor manufacturers to include metadata protocols within their hardware.

### Centralized data management with distributed access

In many sensor networks like GAMUT, physical monitoring infrastructure (e.g., sites and sensors) and their management (e.g., equipment owners and technicians) are distributed across locations/watersheds, personnel groups, and universities. However, server infrastructure and software CI are commonly centralized. This structure is implemented to avoid duplication of server infrastructure and CI personnel in multiple locations. Given available resources, the GAMUT data needed to be centrally housed and managed on server infrastructure at USU, with access to the data provided to many distributed users. An additional requirement was to provide levels of access to the data consistent with what is needed by particular users (e.g., data consumers need read only access, but data managers need the ability to read and write).

Tremendous investment in instrumentation and human resources goes into creating and operating an observatory such as GAMUT. Often underestimated are the related investments in computer infrastructure that must be made to safely store, archive, and back up the data, as well as facilitate data management. Hardware resources that could facilitate consistent data storage, archival, and application of system backups were required.

## Data management and publication workflow

**Fig. 4:**
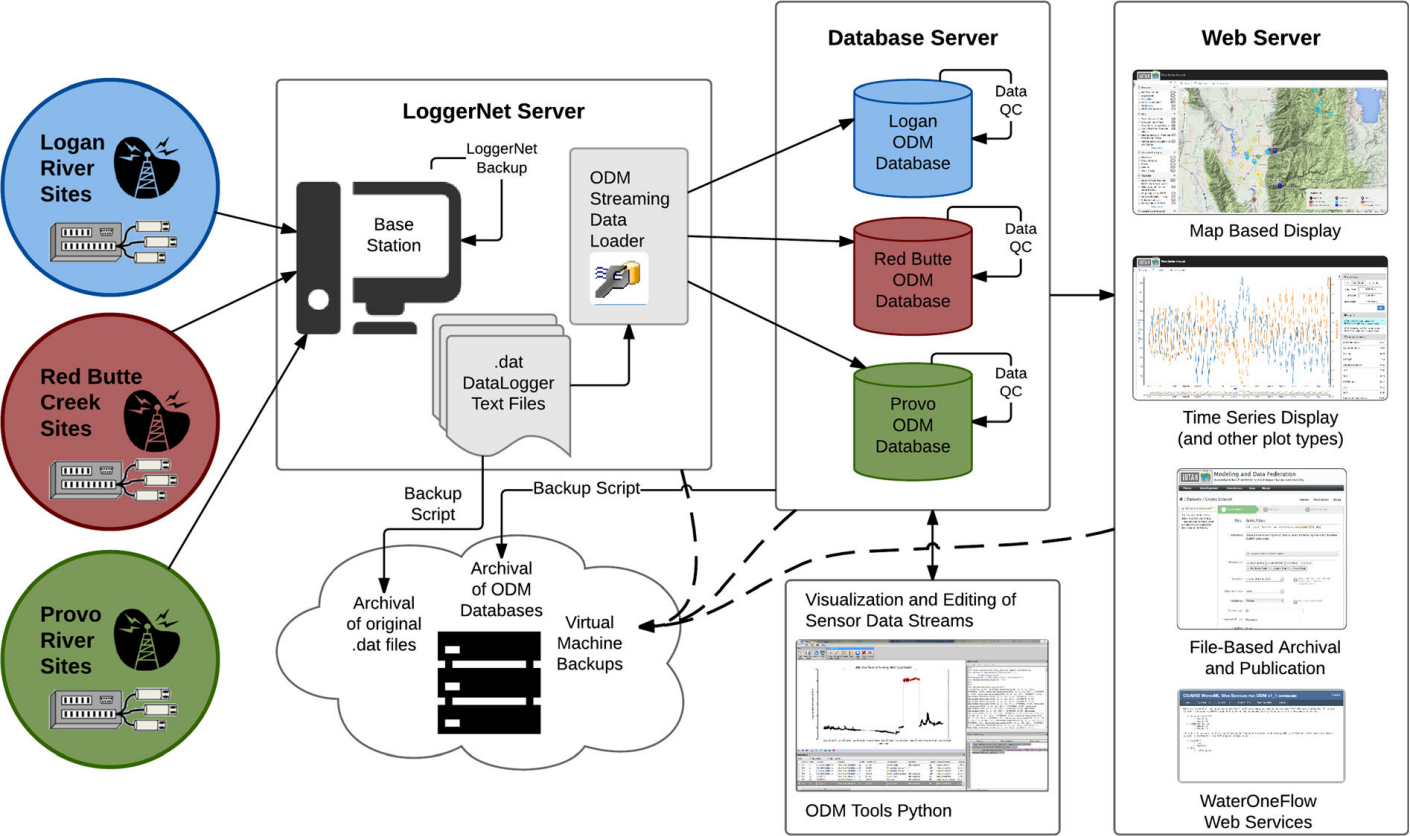
GAMUT sensor data management workflow

To meet the requirements described above, we developed and implemented the workflow shown in Fig. 4. The following sections describe specific components of the workflow. Mason et al. (2014) accurately refer to the data management as the “shepherding” of data from generation to publication, and Rüegg et al. (2014) point out that data management should be a planned process that enhances science rather than an afterthought. Here, we document each step of this data life cycle and the processes undertaken to manage the data. The underlying server architecture we have chosen is described in “Server infrastructure.” From left to right in Fig. 4, the raw data flows from remote field sites to a centralized base station (“Monitoring site design and communications”). Raw data retrieved to the centralized base station are then automatically loaded (“Streaming data loading”) into operational databases on a database server (“Operational databases”). Management of datalogger programs for all remote sites is described in “Managing datalogger programs.” Once loaded, raw data are automatically screened for quality assurance (“Automated quality assurance checks and alerts”) and then post-processed offline for quality control (“Data quality control post-processing”). Both raw and processed data are published using a suite of web applications (“Data publication and sharing”). The data are archived at various stages (“Data archiving”), along with regular backups of the servers that run the workflow. We also describe the implementation of equipment tracking and management (“Equipment management and tracking”).

### Server infrastructure

The GAMUT data management workflow is divided between three virtual servers, each of which is running the Microsoft Windows Server 2008 R2 Operating System. The first server acts as a centralized base station for the GAMUT telemetry network and manages communication and data download from each monitoring site. The second server runs Microsoft SQL Server and hosts the operational databases into which the sensor data are loaded and stored after download. The third is a web server on which several web applications are hosted for sharing GAMUT data over the Internet. Although all of these functions could be performed on a single server, we separated them into three separate virtual machines for security and performance purposes. Because most outside users only need access to the web applications running on the web server, the telemetry base station and database servers can be protected behind additional firewalls with controlled access to credentialed users and limited external exposure to the Internet. The web server, on the other hand, provides unrestricted access to the data via web application user interfaces. Versteeg et al. (2006) also separated server infrastructure based on function to conserve computational resources. This structure meets the requirements described in “Centralized data management with distributed access”.

### Monitoring site design and communications

Each of the GAMUT climate and aquatic sites has a standard suite of sensors installed (Table 1), which are connected to a Campbell Scientific, Inc. datalogger (http://www.campbellsci.com). The datalogger executes the programming logic to operate data collection at the site and provides on-site data storage. The datalogger programs deployed are standardized to sites of each type (aquatic and terrestrial) and new versions of programs are documented. A standard program template is used with site specific modifications (e.g., constants) implemented as needed. This ensures consistency of data collection across sites and watersheds. Details of datalogger program management are provided in “Managing datalogger programs.”

The GAMUT network employs a variety of telemetry connections, including spread spectrum radio frequency, cellular, and internet protocol (TCP/IP) to transfer data from field sites to remote base stations. From remote base stations, communications are made via TCP/IP to the centralized base station at USU. The centralized base station runs Campbell Scientific’s LoggerNet Server software. LoggerNet enables automated communication with field sites, scheduled download of data, delivery of new programs or instructions to a site, and a set of communications diagnostic tools. For most sites in the GAMUT network, scheduled downloads of observations to the LoggerNet Server are made on an hourly basis. The data are contained in comma-separated “.dat” datalogger text files to which new data are appended as they are acquired. This functionality facilitates automation of the workflow (“Automated workflow process”). Although LoggerNet is commonly used and enables significant functionality, other data logging and communication platforms exist, including recent efforts toward the development of open source data logging platforms (e.g., Abraham and Li 2014; Ferdoush and Li 2014). As long as retrieved data are stored in table-based, delimited text files, a common denominator with many such systems, the subsequent steps in the workflow can be used.

### Streaming data loading

To automate the process of loading data from datalogger files to relational databases, we implemented the Streaming Data Loader, a component of the CUAHSI HIS HydroServer software stack (Horsburgh et al. 2011). The Streaming Data Loader can be configured to load any number of table-based text files (i.e., files generated by dataloggers) to databases that implement the CUAHSI HIS ODM (Horsburgh et al. 2008). In the initial setup for loading a file, a data manager uses the Streaming Data Loader to specify the relevant metadata (e.g., site, variable, method) for each column in the file to be loaded to the database, where each column represents a time series of data for a single variable. When it is executed, the Streaming Data Loader opens the datalogger text file, checks the latest date for which data were collected, compares that date to the latest date for the data series in the ODM database, and loads any new data to the database. When new sites come online or when additional variables are added, files or columns are added using the configuration file or the Streaming Data Loader interface.

The Streaming Data Loader is configured to run automatically as a Windows Task using the Windows Task Manager on the LoggerNet Server. As an automated Windows Task, the Streaming Data Loader can be set to run at any frequency. For the GAMUT network, we have established hourly data loads corresponding to the hourly acquisition of new data from the dataloggers so that new data are available in the operational database in near real time. The implementation of the Streaming Data Loader is essential to meet the requirement for automation of the data management workflow (“Automated workflow process”) as well as make data available in near real time (“Data available in near real-time via the Internet”).

In the GAMUT network, several aquatic sites were co-located with existing stream gages maintained by the US Geologic Survey (USGS) and the Central Utah Water Conservancy District (CUWCD). In order to take advantage of this existing instrumentation and data collection, we determined to ingest these data into the GAMUT ODM databases. These agencies output their data to delimited text files that are updated as new observations are made and are accessible via the Internet. The Streaming Data Loader can access web-based files, so we have mapped these data files and are loading them into the databases along with data from GAMUT sensors.

### Managing datalogger programs

**Fig. 5:**
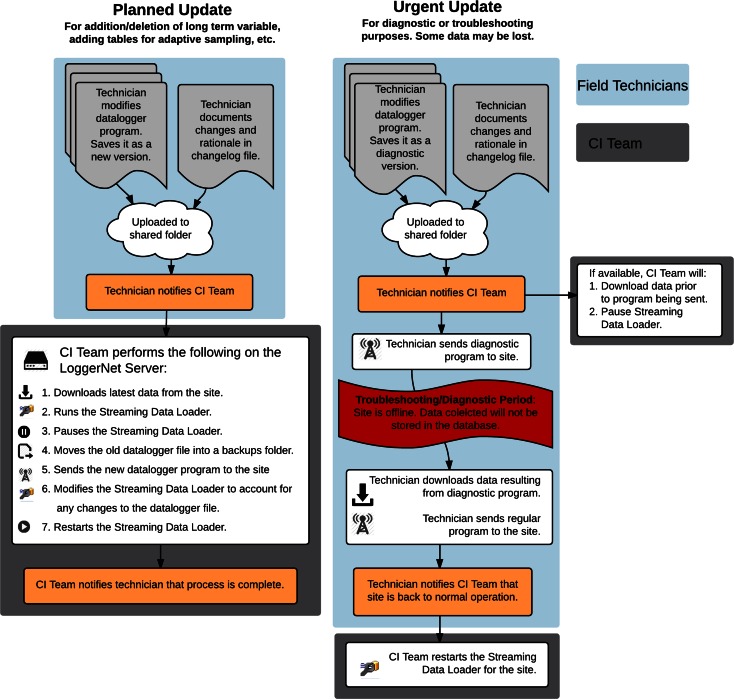
Process for making changes to datalogger programs to avoid data loss

The datalogger programs for each monitoring site in the GAMUT network are updated periodically to reflect addition or removal of sensors, modification of programming logic to improve results, troubleshooting of malfunctions or failures, etc. Each time a new program is sent to a Campbell Scientific datalogger, all of the data in the tables stored in the datalogger’s memory are erased. This posed a problem for our data collection system—if the most recent data stored on the datalogger were not retrieved prior to a new datalogger program being sent, data values would be lost causing gaps in the data series. To avoid this to the extent possible, we devised a procedure for managing updates to the sites’ datalogger programs (Fig. 5). It includes scenarios for both planned updates to datalogger programs and potentially urgent updates for diagnostic or troubleshooting purposes. Procedures for each of these scenarios were designed to minimize down time and data loss at monitoring sites. These procedures are related to keeping data available in near real time (“Data available in near real-time via the Internet”) as well as centralized data management (“Centralized data management with distributed access”).

Under the planned update scenario, a technician modifies a program and documents the rationale for the modifications in a changelog file. The revised program is then uploaded to a shared folder, and the iUTAH CI Team is notified that a new program is available. The CI team then sends the new program to the site, following the steps shown in Fig. 5 to ensure that no data are lost. Occasionally, urgent updates to datalogger programs are needed for troubleshooting or diagnostic purposes (e.g., sensor or equipment malfunctions). Under this scenario, the technician connects to the site of interest and sends the diagnostic program. When diagnostics are complete, the technician downloads data resulting from the diagnostics program and loads the previous program onto the datalogger to resume normal operations. It is acknowledged that some data loss may occur for the period during which the site is in diagnostics mode and for a short period beforehand if the latest values are not retrieved prior to the diagnostics program being sent.

### Operational databases

As shown in Fig. 4, three ODM database instances were deployed to store the streaming sensor data, one for each watershed. Separate databases were implemented rather than a single, larger database to provide an additional level of security and granularity. This is beneficial for limiting access to users who only need to access the data for one of the watersheds as well as being able to easily partition between watersheds for web applications. The GAMUT databases were implemented on the database server using the Microsoft SQL Server 2012 relational database management system (RDBMS). The databases provide transactional access to the data using Structured Query Language (SQL), which facilitates data loading by the Streaming Data Loader as well as management and visualization. Microsoft SQL Server met our requirements for database infrastructure (“Centralized data management with distributed access”), but as it is not open source, other monitoring or research groups might choose to implement another RDBMS (e.g., MySQL, PostgreSQL). Most of the workflow components are compatible with these RDBMS.

Within the CUAHSI HIS, ODM was designed to store observational data along with complete metadata to facilitate unambiguous interpretation of data (Horsburgh et al. 2008), a need described by Rüegg et al. (2014) to facilitate data reuse and increase the value of datasets beyond the initial objective. Metadata stored in ODM include details about what data were collected (variable), how data collection was conducted (method), where the observations were made (site), who collected the data (source), as well as temporal information. ODM also includes controlled vocabularies for many of the attributes of data to ensure that data are described consistently across ODM instances (Horsburgh et al. 2014). Using ODM increases the sustainability and interoperability of the data because ODM is established, well-documented, and has existing software available for loading data (e.g., the Streaming Data Loader) and for publishing the data in the databases with the CUAHSI Water Data Center. Using ODM also helps meet requirements for “Data versioning and archival”.

### Automated quality assurance checks and alerts

Once the data are loaded into ODM databases, automated checks are performed to alert the iUTAH watershed technicians of data anomalies or potential problems in the new data. These alerts are implemented as stored procedures within the ODM databases. They regularly scan new data, check whether certain conditions have been met, and trigger e-mail messages to the appropriate technicians when anomalous conditions are detected. Implementation of these procedures addresses the needs described in “Automated quality assurance”. Alerts that have been implemented include battery voltage checks (to circumvent power failures), variable-specific range checks (indicating a potential sensor failure), data value persistence checks (indicating a stuck sensor or potential sensor failure), variable-specific value change threshold checks (indicating a potential sensor failure or unusual site conditions), and data currency checks (indicating a sensor, power, telemetry, or data loading failure). The thresholds for each of these checks can be customized for specific variables and sites, and additional checks can be added as needed. Source code for the stored procedures we developed is freely available in a GitHub repository for other ODM users.

### Data quality control post-processing

**Fig. 6:**
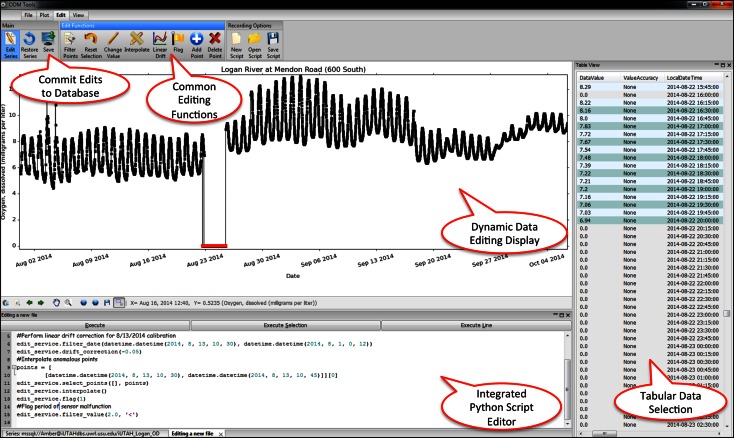
Screenshot of ODM Tools Python while performing quality control edits. Editing steps selected from the editing toolbar are automatically recorded to a Python script shown at the bottom of the main window

To remove anomalous values, correct for sensor drift, add qualifier flags to data points, and arrive at “approved” versions of the time series from the GAMUT sensors, we developed a software tool for post-processing the sensor data. ODM Tools Python is an open source software application that facilitates query, export, visualization, and quality control editing of data stored in an ODM database (Horsburgh et al. 2015). ODM Tools Python allows the user to query and export data series and associated metadata, plot and summarize a data series, generate a new data series, and perform quality control editing. ODM Tools Python also has the ability to plot multiple data series with several plot types (which is important in post processing time series data for quality control), runs on multiple platforms (Windows, Linux, and Mac), supports multiple RDBMS (Microsoft SQL Server, MySQL, and PostgreSQL), and enables automated scripting of edits through an integrated Python script editor and console. Figure 6 shows the ODM Tools Python GUI while performing and scripting edits on a data series.

The ability to record editing steps was a principle objective motivating the development of ODM Tools Python. Data quality control can be a subjective process where individuals starting with the same data series could arrive at different conclusions. Using a script to record edits allows users to compare, review, and even modify the steps that have been taken to perform quality control. In ODM Tools Python, edits are automatically recorded to a script that can then be saved for future reference and execution. In this manner, the steps taken to arrive at an approved, quality-controlled data series are traceable and reproducible. For the GAMUT Network, the Python scripts generated by ODM Tools Python serve as the record of edits made to the raw data and can be executed at any time to regenerate the quality-controlled dataset, meeting the requirement for “Standardized and traceable quality control”.

Though the term “editing” is used here to refer to data adjustments, the standard for the GAMUT network is to perform all edits on a copy of the raw data. ODM Tools Python connects directly to an ODM database to access data. As a user performs edits on a raw data series, the changes are made on a copy of the raw data that is stored in the local memory of the data analyst’s computer. When edits are complete, the revised data series with any data qualifying comments can then be saved to the same database with a new method, quality control level, or variable to distinguish it from the raw data as a separate version. In this way, the original sensor data are maintained.

### Data publication and sharing

Storing the GAMUT data in ODM databases provided an operational structure that facilitated implementation of web applications to publish the data on the Internet. We implemented multiple mechanisms for online data access and sharing that meet the requirement for making “Data available in near real time via the Internet”. First, we deployed CUAHSI HIS WaterOneFlow web services, which connect directly to the ODM databases to publish and deliver the GAMUT data in WaterML format in response to web service requests (Zaslavsky et al. 2007). We registered the WaterOneFlow web services with the CUAHSI Water Data Center to ensure that the data are discoverable and accessible through the Water Data Center and via the CUAHSI HIS HydroDesktop software (Ames et al. 2012).

Establishing WaterOneFlow web services also enabled us to develop additional software that uses the web services to access the GAMUT data. For example, we developed a new, web-based tool for data visualization and dissemination called the Time Series Analyst (TSA) (http://data.iutahepscor.org/tsa/). Web services also open the possibility for other researchers or groups to develop and implement specialized interfaces for data access for various audiences (important functionality suggested by Mason et al. 2014). Furthermore, individual researchers can write code using R, Python, Matlab or other scripting languages to retrieve data from the web services directly into a coding or analytical environment. The web services act as a mechanism for providing read-only access to the data. This serves to protect the integrity of the underlying databases by limiting direct database access to experienced data managers.

**Fig. 7:**
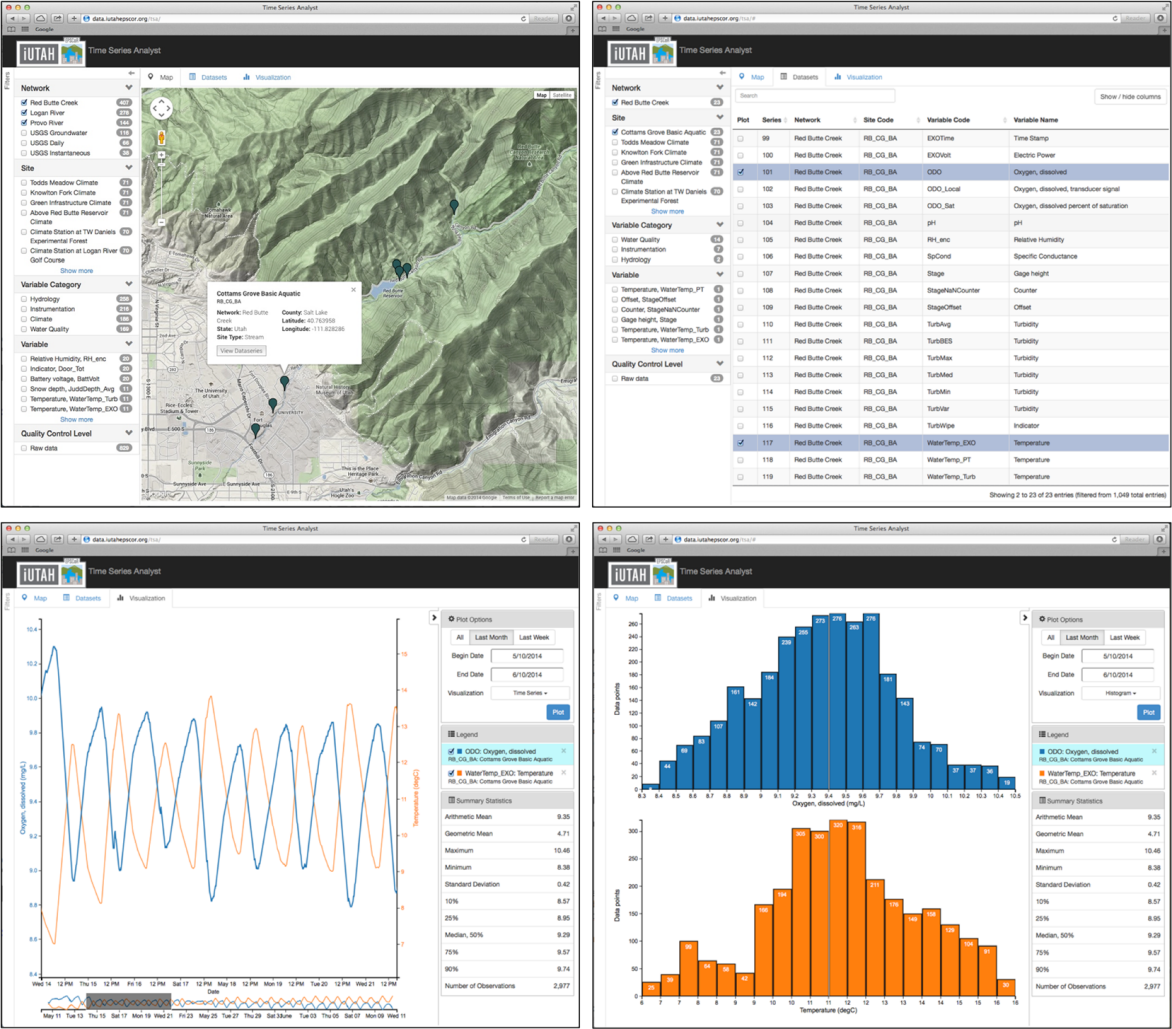
Screenshots of the mapping tab, dataset selection tab, and visualization tab of the Time Series Analyst web interface

The TSA is a simple yet powerful tool that extends the reach of the GAMUT data by providing visualizations of the data to a variety of users in a web browser. The TSA consists of a Google Maps-based interface to spatially visualize the locations of the GAMUT sites, a selectable list of data series available at each site, and a plotting interface. These three components are presented as tabs alongside a panel of search facets by which the available data series can be filtered to facilitate discovery and narrow selection of data series for visualization or download. Locating the monitoring sites on a map provides context for data selection and spatial reference for data interpretation. Figure 7 shows screenshots of the TSA user interface.

The TSA retrieves data for visualization from the GAMUT WaterOneFlow web services. The GAMUT ODM databases are queried daily using a stored procedure to generate a catalog table that contains a list of available data series, time periods for which data are available, and a web service URL for each time series that can be used to retrieve a WaterML file containing the data. The catalog also includes metadata attributes that are used as the facets by which the time series can be filtered. Because WaterOneFlow web services have also been deployed for many other data sources, including the USGS National Water Information System, we are able to include references to USGS instantaneous, daily value, and groundwater sites within the GAMUT watersheds in the TSA catalog and provide access and visualization alongside the GAMUT data. The TSA software is open source, and could be deployed for use with time series of data from any WaterOneFlow web service.

**Fig. 8:**
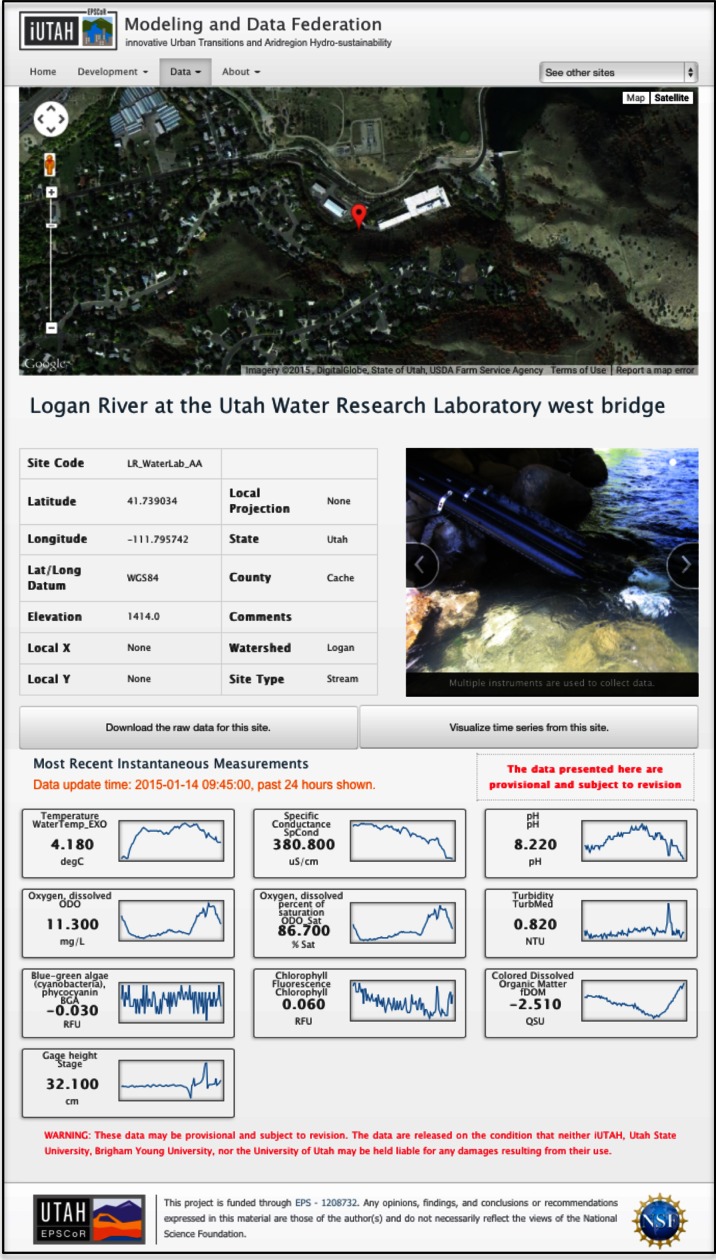
Screenshot of the webpage for an individual monitoring site within the GAMUT network showing site information and recent conditions

In addition to the TSA, we have deployed a static webpage as a GAMUT homepage (http://gamut.iutahepscor.org) with web pages for each watershed and monitoring site in the GAMUT network. The site pages include site metadata, a photo gallery, and spark line plots for variables of interest to indicate current conditions and trends over the past 24 h (Fig. 8). The spark lines link directly to the corresponding visualization in the TSA to facilitate further exploration. The individual site pages serve as a landing page and initial entry point for data access and visualization by new or novice users.

Finally, both the raw and quality-controlled time series from the GAMUT network are formally published within the iUTAH Data Publication System (http://repository.iutahepscor.org). This publication system is based on CKAN, which is an open source data publication system (http://ckan.org). It was deployed to support publication of the heterogeneous datasets that are being generated, collected, or that are of interest to the iUTAH community and enables publication and archival of finalized, file-based datasets. In the case of the continuous GAMUT data, annual, file-based snapshots are published. For the raw data, a single file is produced per site for each year containing data for all variables collected at a site. The file for the current year is updated daily until the end of the year when the file is finalized. The quality-controlled time series are published in a similar manner but are updated periodically as quality-controlled data become available. This functionality helps address the need for “Data versioning and archival”.

Snapshots of both raw and quality-controlled data are archived within the data publication system as comma-separated, ASCII text files. They provide an additional data access mechanism for users who want to work with many variables at one time, rather than accessing them individually through the web services or TSA. The text files can easily be imported into many data visualization or analysis software packages. Each dataset published within the data publication system is assigned a unique and persistent URL as an identifier and a citation that is consistent with DataCite’s style recommendations (https://www.datacite.org/services/cite-your-data.html).

### Equipment management and tracking

**Fig. 9:**
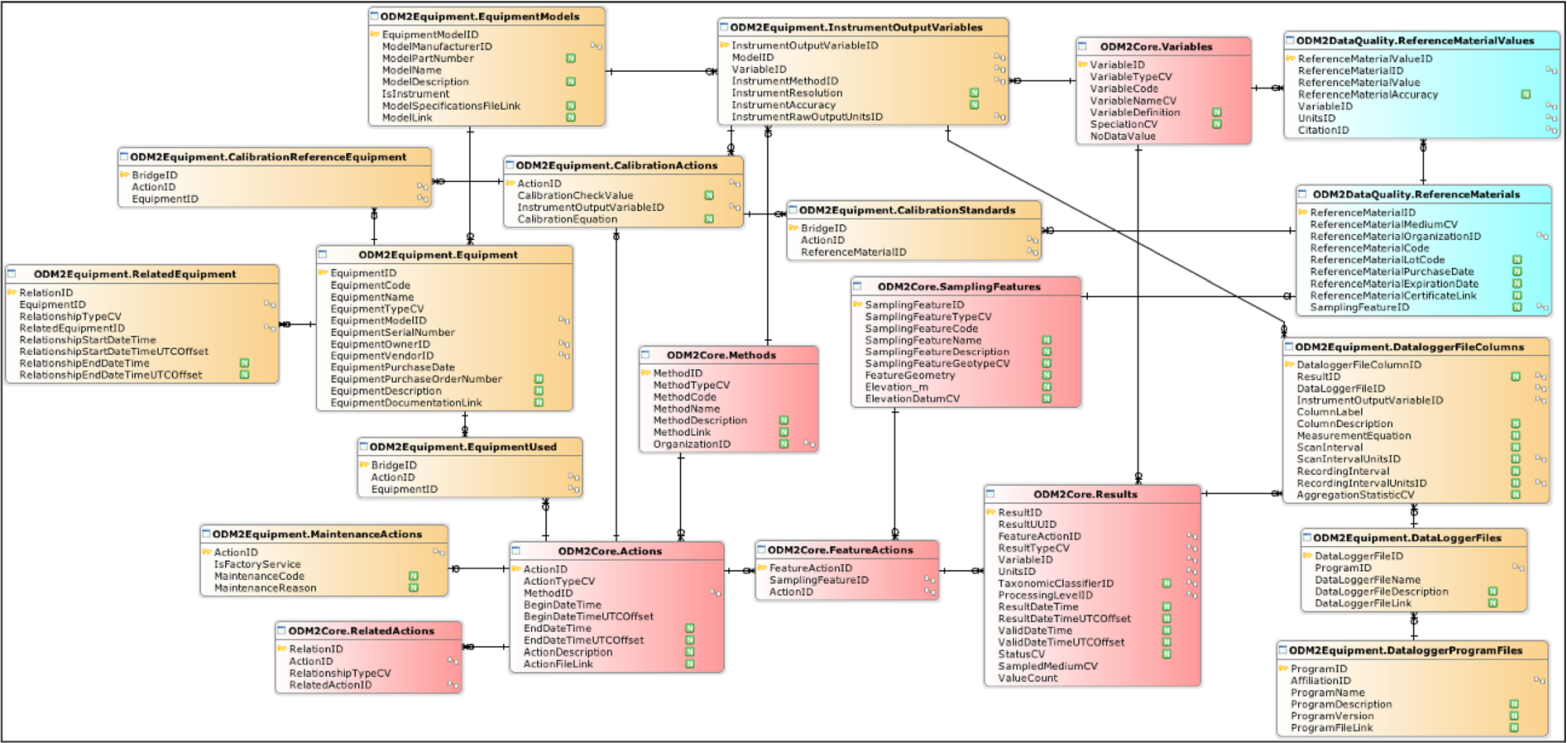
Data model for equipment management

To address the storage and tracking of equipment and activities, we developed a data model to house the records and a web interface for field technicians to enter and access the information. The underlying data model includes entities to store equipment and related details, actions that include site visits, equipment deployments, instrument calibrations, and factory service maintenance, information on datalogger programs, and details on calibration standards and equations (Fig. 9). This equipment database was designed to extend the ODM databases in which the GAMUT observational data are stored so that the equipment management information can be directly related to the observational data, a requirement described in “Data linked to field information.”

**Fig. 10:**
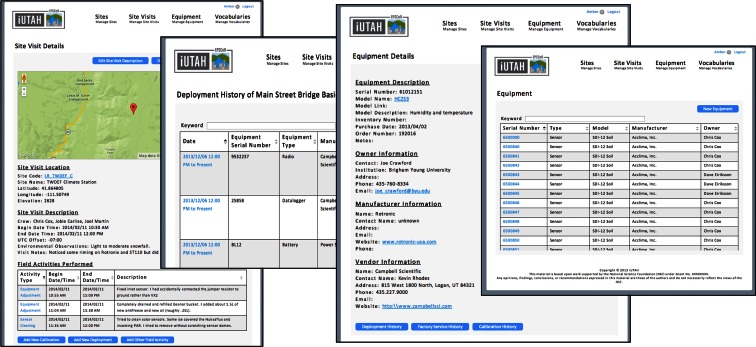
Screenshots of the web interface for the GAMUT network equipment management database

Screenshots of the web interface to access and modify the underlying data model are shown in Fig. 10. The GAMUT field technicians use the web interface to enter information about individual pieces of equipment, deployments and calibrations, and factory service maintenance. The web application permits the technicians to query and view information such as the equipment deployed at a particular site, the calibration history, factory service history, and deployment history of a sensor, and notes and additional information on site visits and site maintenance. These details can be accessed simultaneously while conducting quality control on the observational data. Note that as part of quality assurance, this step is not directly depicted on the data management workflow in Fig. 4, but contributes to the quality of the data that are collected.

### Data archiving

As indicated in Fig. 4, our workflow employs multiple levels of backup so that there is redundancy in the archival process, helping address archival and automation requirements (“Data versioning and archival” and “Automated workflow process”). At the most basic level, we retain some local data storage on the dataloggers at each monitoring site. This is not a long-term data store, as older data are eventually overwritten as newer data are collected. However, if our central data management system goes offline, data collection and recording will continue, and the data can be retrieved and imported as soon as the central system comes back online. Next, LoggerNet has a built-in backup utility that copies the configuration of the LoggerNet network and all data files to a backup file on a weekly basis. In the case of a failure of the LoggerNet Server, this configuration could be imported to a new Loggernet server and the network and data transfer would proceed.

The text files containing data values that are downloaded from each remote site are also archived. This occurs daily via a Windows backup script for the active datalogger files that are being updated in real time. In addition, if changes are made to the datalogger program that could result in a new file being initiated, the old file is copied to an archive folder, which is also backed up. By taking these steps, we ensure that the original data files are always available as downloaded from the monitoring site. These files could be used to fully reconstruct the record of data if needed. A SQL Server backup script is executed weekly to archive the operational databases as file-based backups. In the case of a database failure, the database backups could used to restore the database. Any data gaps since the last database backup would be automatically filled the next time the Streaming Data Loader runs.

Finally, all three virtual servers over which the workflow is distributed are scheduled for daily incremental backups and weekly full backups using the backup capabilities of the virtualization software. This step ensures that in the case of a failure of the physical hardware on which the virtual machines are hosted, the complete virtual machine could be moved to a new physical host with no data loss and minimal loss in services. All of the backups described here are copied from USU to an offsite data store at the UofU for redundancy and to insure against a catastrophic event at USU.

## Conclusions

The workflow and CI described here and the tools we have implemented serve to meet the requirements for data storage, management, and sharing that we identified for the GAMUT sensor network and effectively transfer data from remote field sites to ultimate end-users. Though the multi-watershed, multi-institution nature of GAMUT is somewhat unique, it is becoming more common for large, interdisciplinary groups to work together in developing environmental observatories with similar challenges and requirements for data management. We anticipate that these needs are comparable for other research groups and networks.

We have made use of existing open source, community-developed, environmental CI by adopting components of the CUAHSI HIS, while expanding and further developing tools to enhance functionality. This served to ensure that the GAMUT data are discoverable and accessible through the CUAHSI Water Data Center and met our needs for a platform on which we could build customized components (like the TSA) for enabling enhanced data access and visualization. The components of the workflow that we have developed could be used by other environmental observatories. The workflow is modular, so an observatory could deploy the full suite of elements in a similar workflow, eliminate certain components if deemed unnecessary, or include additional components given network-specific needs. For example, new applications may be developed to interface with data stored in an ODM database or accessible via web services. Much of the workflow could also be adapted to other operating systems or software programs depending on user expertise. For example, ODM and the WaterOneFlow web services are available with deployments for both Windows and Linux servers.

The workflow that we have developed standardizes data management across the three watersheds, institutions, and related personnel. It accounts for protection and backup of the operational system and the observational data. It makes data post-processing steps traceable and reproducible, and it formally stores information related to field activities and management of monitoring infrastructure. Many of the data transfer and data management processes are automated, which should be an objective for all environmental observatories. There are still steps that require the attention and expertise of field and data technicians (e.g., initial metadata entry and mapping of streaming data files, entry of events for equipment management, updating of datalogger programs), but our capacity to collect, transfer, and process data is enhanced. Our workflow also helps curate the data and make it sustainable. The data are stored in recognized formats, and we are using community standards, open-source software, and web-based access to promote reusability and discovery of the data. Finally, the implementation of a number of steps toward data quality assurance and quality control serve to ensure that the data collected are of the highest possible quality.

## Software availability

The software programs described herein are available via open source code repositories. The CUAHSI HIS HydroServer software stack, including ODM, the Streaming Data Loader, and WaterOneFlow web services are available on the HydroServer Codeplex website and code repository (http://hydroserver.codeplex.com). Other applications developed at USU are available on GitHub: ODM Tools Python (https://github.com/ODM2/ODMToolsPython), stored procedures for automated data quality assurance (https://github.com/UCHIC/iUtahUtilities/tree/master/src/GAMUTDataAlerts), TSA (https://github.com/UCHIC/WebTSA), equipment management website (https://github.com/UCHIC/ODM2Sensor), and the GAMUT watershed and individual site static webpages (https://github.com/UCHIC/iUTAHData).
